# Electrophysiological function in eyes with reticular pseudodrusen according to fundus distribution

**DOI:** 10.1371/journal.pone.0203146

**Published:** 2018-08-29

**Authors:** Mingui Kong, Jaemoon Yoon, Don-Il Ham

**Affiliations:** 1 Hangil Eye Hospital, Incheon, Korea; 2 Department of Ophthalmology, Catholic Kwandong University College of Medicine, Incheon, Korea; 3 Seobusan Eye Clinic, Busan, Korea; 4 Department of Ophthalmology, Samsung Medical Center, Sungkyunkwan University School of Medicine, Seoul, Korea; University of Alabama at Birmingham School of Medicine, UNITED STATES

## Abstract

Reticular pseudodrusen (RPD) could be present not only in the posterior pole but extramacular area also as a confluent morphological pattern. Thus RPD can be classified by the fundus distribution for the assessment of visual prognosis. The electrophysiological function in eyes with reticular pseudodrusen (RPD), showing various fundus distribution were evaluated using full-field electroretinogram (ERG). Retinal distribution of RPD was divided into three types (localized, intermediate, and diffuse) according to the extent of involvement of retinal areas by fundus photograph montages. RPD were present with the diffuse type in 21 eyes (25.6%), with the intermediate type in 30 eyes (36.6%), and with the localized type in 31 eyes (37.8%). The average age was 74.76 ± 4.52 (range, 65–81) years in the diffuse type, 72.47 ± 9.13 (range, 55–91) years in the intermediate type, and 70.26 ± 7.77 (range, 61–89) years in the localized type. The mean amplitudes of the scotopic rod response, scotopic maximal combined response, oscillatory potentials (OP), photopic cone response, and 30Hz cone flicker response were more decreased in the diffuse, intermediate, and localized types in order, except for the photopic cone a-wave response. The diffuse type showed reduced amplitudes of ERG responses than the normal control group under all testing conditions except for the photopic a-wave response, and differences were statistically significant with the age restriction and adjustment methods (Bonferroni-corrected *P* < 0.05). The mean implicit times of ERG responses were significantly delayed in the diffuse type in the photopic b-wave. (Bonferroni-corrected *P* < 0.05). Extensive retinal involvement of RPD correlates with severely reduced electrophysiological retinal function. This acquired form of decreased electrophysiological function should be regarded as different from those of hereditary retinal degeneration.

## Introduction

Reticular pseudodrusen (RPD) were first described in 1990 as “pseudodrusen visible in blue light”,[1] and many findings reported thereafter indicated that RPD differ from soft drusen in many aspects.[2–4] RPD have very unique features, such as characteristic multiple fundus lesions with reticular network, deposits under the sensory retina and above the retinal pigment epithelial (RPE) layer in spectral-domain optical coherence tomography (SD OCT), and different findings from soft drusen in near-infrared, fundus autofluorescence (FAF) imaging, fluorescein angiography (FA), and indocyanine green angiography (ICGA).[2–4]

Although the number of published papers has increased, much is still unknown about RPD, including its distribution in the fundus. Most of the previous reports described the distribution of RPD in the posterior pole, and curiously, several reports commented on the presence of extramacular and peripheral RPD.[4–8] We previously reported that RPD are present in the macula area and at the periphery. [9] RPD with diffuse distribution can show a confluent morphological pattern and a high prevalence of late age related macular degeneration (AMD), and RPD can be classified by the fundus distribution for the assessment of visual prognosis. However, retinal function in RPD is unclear. It is probable that the presence of RPD is associated with abnormal retinal function, because deposits are located just beneath the sensory retina with partial disruption of photoreceptor outer segments,[4] and retinal distribution is often widespread with high density.[5] However, in a previous study about the retinal function using multifocal electroretinography in eyes with RPD, no definite influence was observed on electrophysiologic activity in areas with RPD.[10] On the contrary, it has been also reported that RPD progression caused outer retinal atrophy and impairments in dark adaptation.[11, 12]

Better understanding of the functional characteristics of RPD would lead to the use of more reasonable and acceptable standard examination methods, and grading systems for evaluation. In this report, we evaluated the relation between fundus distribution and the electrophysiological function of retina in eyes with RPD.

## Subjects and methods

### Subjects

The medical records of patients previously diagnosed as RPD between January 2003 and April 2010 at the retinal service of Samsung Medical Center, Sungkyunkwan University, Seoul, Korea, were reviewed. This study received institutional review board (IRB) approval through the Samsung Medical Center Institutional Review Board and it was conducted in accordance with the ethical standards of the Declaration of Helsinki. All patients' information was anonymized and the IRB waived the requirement for informed patient consent due to the retrospective nature of this study. Forty two patients with RPD (82 eyes) and 21 healthy subjects (42 eyes) were enrolled in the study. Patients with an ocular disease that might affect the results of the full-field electroretinogram (ERG), such as moderate-to-severe non-proliferative diabetic retinopathy (NPDR), PDR, optic neuropathy, retinal vascular occlusion, and retinal detachment, were excluded. In the normal control group, no subject had systemic or ocular diseases, except mild to moderate lens opacities, and mild and focal pigmentary disturbances not associated with drusen on fundus examination.

### Diagnosis, imaging, and ERG

Each patient had undergone ocular examinations including slit lamp examination, and funduscopy. All patients had undergone at least one imaging test, including color fundus photography with blue channel examination, red free (RF) photography, near-infrared (IR) photography, fundus autofluorescence (FAF) imaging, spectral domain optical coherence tomography (SD OCT), fluorescein angiography (FA), and indocyanine green angiography (ICGA). Fundus color photographs were taken with a model IX50 camera (Topcon, Paramus, NJ), and were viewed in the Topcon ImageNet program (version 2.56, Topcon) for blue channel examination, as described elsewhere.[3, 4] Red free photography, near-infrared photography, FAF imaging, SD OCT, FA and ICGA were performed using a Spectralis HRA+OCT (Version 1.5.2.0; Heidelberg Engineering, Heidelberg, Germany), and viewed with Spectralis Viewing Module 5.3.2.0 software (Heidelberg Engineering). The Spectralis instrument allowed for topographic correlation between SD OCT and near-infrared images.

Diagnosis of RPD was based on the following appropriate findings: 1) multiple yellowish white lesions with a reticular network in color photographs and blue channel examinations, 2) light, interlacing network in red free imaging, 3) hyporeflectant lesions with mild background hyperreflectance in near infrared imaging, 4) hypofluorescent lesions against a background of mild hyperfluorescence in FAF imaging, 5) subretinal deposits in SD OCT, and 6) hypofluorescent lesions in the mid or late phase of ICGA. FA was used as an ancillary test for the differential diagnosis. FA is useful to detect cuticular drusen, sub-retinal pigment epithelial deposits showing characteristic "stars-in-the-sky" appearance in FA, which sometimes mimic RPD. Images of each eye were assessed independently by two retinal specialists (J.Y. and D.H.). Agreement between the assessors was necessary for the diagnosis of RPD.

Fundus montages of color photographs were used for the analysis of RPD distribution. Montages were created by merging five color pictures, including the central macular field, and four adjacent fields (superior, inferior, temporal, and medial to the central field), taken with a 50 degree-angle camera (IX50, Topcon), as reported previously.[9] Fundus montages showed the distribution of RPD up to the mid-peripheral fundus, which were classified into three types according to the distribution; localized, intermediate, and diffuse (S1 Fig).[9]

For ERG testing, pupils were dilated to at least 7 mm diameter with 0.5% tropicamide, and both corneas were anesthetized with proparacaine hydrochloride 0.5% before insertion of Burian-Allen electrodes (Hansen Instruments, Inc., Iowa City, IO). Ganzfeld full-field ERGs were performed on both eyes according to International Society of Clinical Electrophysiology of Vision (ISCEV) standards (RETIscan system, Roland Consult, Wiesbaden, Germany). The scotopic rod and scotopic maximal ERGs were recorded after a minimum of 20 minutes dark adaptation, and the photopic 30 Hz flicker and single flash photopic ERGs were recorded after 10 minutes of adaptation to a background light. Oscillatory potentials (OP) were recorded simultaneously with the conventional ERGs. The mean amplitude and implicit time of the a-waves, b-waves, and the OP were measured on both eyes of each subject. ERG responses were compared between three distribution types, and normal controls.

### Statistics

The data obtained were analyzed using independent t test, chi-square test, Kruskal-Wallis rank sum test, Fisher's exact test. Partial spearman correlation coefficient analysis with Bonferroni’s correction was used to describe the relationship between distribution types and control group. Age and sex were adjusted, and a *P* value < 0.05 was considered to be significant. The statistical analyses were performed with R software version 2.11.1 (R Foundation, Vienna, Austria).

## Results

### ERG responses and distribution types

Eighty six eyes had full-field ERG performed according to the ISCEV protocol. Forty two patients (82 eyes) were female and two patients (4 eyes) were male. Among them, 4 eyes of two young female patients (40 and 44 years of age in the intermediate and localized type, respectively) were excluded, and only ERG data from subjects 50 years of age or older were used to minimize the aging effect on ERG response in comparison. Finally, 82 eyes of 42 patients were used for the ERG analysis. All patients had color fundus photography and blue-channel examination, and 92.7% of patients had more than two imaging tests; 82.9% had SD OCT, 85.4% had infra-red imaging tests, and 76.8% had FAF imaging (Table 1).

**Table 1:** Methods for detecting reticular pseudodrusen.

Imaging Mehod	% of Eyes
CFP + Blue Channel Examination	100%
SD OCT	82.9%
IR	85.4%
FAF	76.8%
RF	90.2%
FA	59.8%
ICGA	19.5%

CFP: color fundus photo; SD OCT: spectral domain optical coherence tomography; IR: infrared images; FAF: fundus autofluorescence images; RF: red free images; FA: fluorescein angiography; ICGA: indocyanine green angiography

Ten patients (23.8%) had a history of diabetes; Four eyes of two patients had mild NPDR, and others had no signs of diabetic retinopathy. Seventeen patients (40.5%) had a history of hypertension with no severe hypertensive retinopathy. There were no cuticular drusen eyes found in multimodal imaging tests.

The average age was 72.2 ± 7.77 (55–91) years; 74.76 ± 4.52 (65–81) years in the diffuse type (21 eyes of 11 patients), 72.47 ± 9.13 (55–91) years in the intermediate type (30 eyes of 16 patients), and 70.26 ± 7.77 (61–89) years in the localized type (31 eyes of 17 patients). The age of the control group was 65.19 ± 10.44 (51–91). Although the mean ages of RPD types were significantly greater than that of the control group (Independent t test, *P* < 0.001), no statistical differences were found among RPD groups (Kruskal-Wallis rank sum test, *P* = 0.067). In the control group, 11 subjects were female (52.4%), and the proportion was significantly different than that in the RPD group (Chi-square test, *P* < 0.001) (Table 2).

**Table 2:** Characteristics according to the distribution type of RPD.

Distribution type	Control	Localized	Intermediate	Diffuse	P-value
Number of Eyes (patients)	42 (21)	31 (17)	30 (16)	21 (11)	N/A
Sex (F:M)	11:10	15:2	16:0	11:0	N/A
Mean age (range, years)	65.19±10.44 (51–91)	70.26 ± 7.77 (61–89)	72.47 ± 9.13 (55–91)	74.76 ± 4.52 (65–81)	0.017
AMD sings other than RPD (eyes)	0	21	24	18	0.281*

RPD: reticular pseudodrusen; AMD: age related macular degeneration

* comparison among three RPD groups.

**Fig 1:**
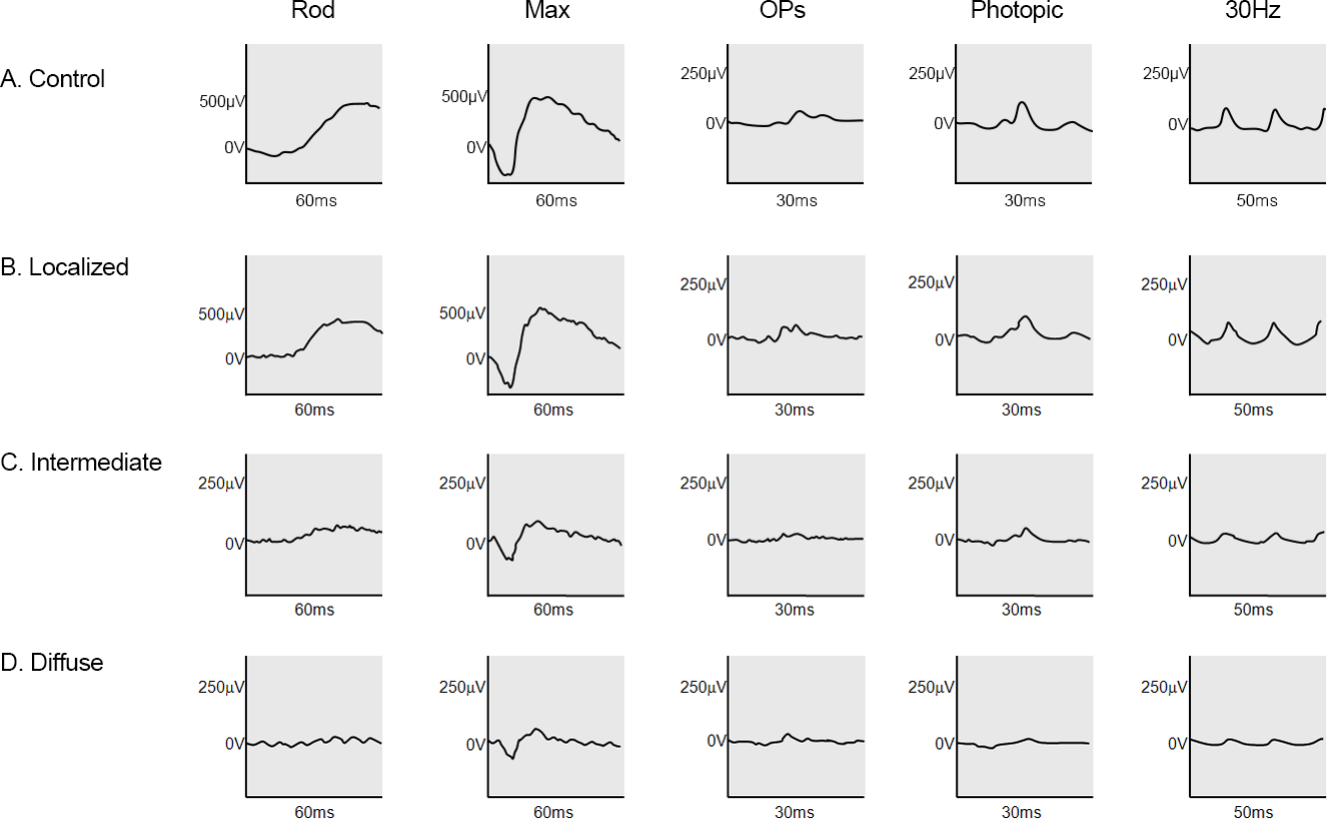
Typical electroretinogram (ERG) responses in the normal controls and three distribution types of reticular pseudodrusen. (A) Normal ERG responses (**B)** The localized type showed almost normal ERG responses. (**C**) The intermediate type showed variously decreased ERG responses with relatively more decreased scotopic responses. (**D)** The diffuse type showed severely decreased ERG responses.

**Fig 2:**
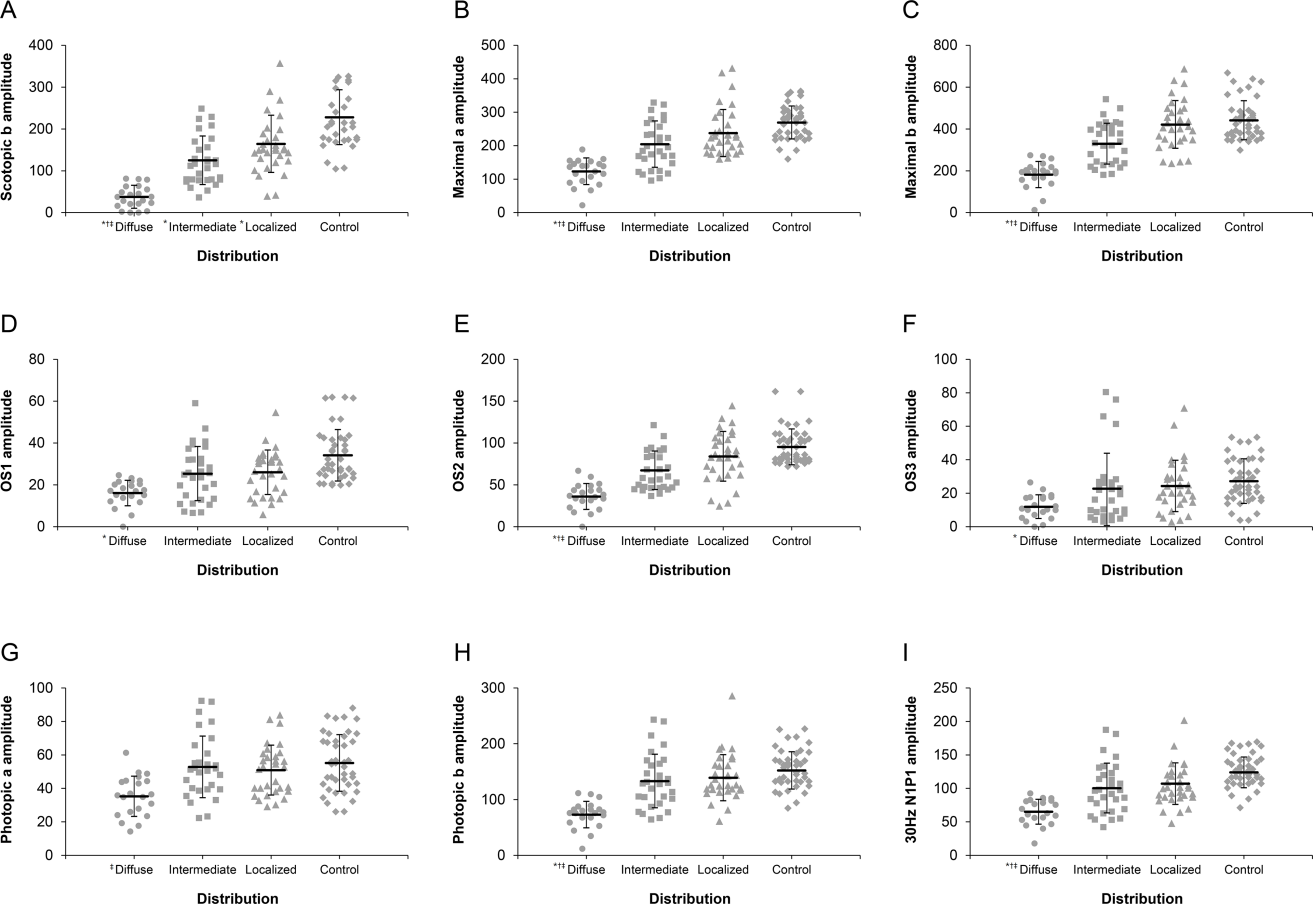
Comparison of electroretinogram (ERG) amplitudes among the three distribution types of reticular pseudodrusen (RPD) and the normal control (NC). (**A)** Statistically significant differences were observed among the three types, and the NC in the mean scotopic b amplitude, except between the intermediate and localized type. (**B)** The mean maximal a amplitude of the diffuse type was significantly reduced compared to those of other types and the NC. (**C)** The mean maximal b amplitude of the diffuse type was significantly reduced compared to those of other types and the NC. (**D)** The mean oscillatory potential (OP)1 amplitude of the diffuse type was significantly reduced compared to that of NC. (**E)** The mean OP2 amplitude of the diffuse type was significantly reduced compared to those of other types and the NC. (**F)** The mean OP3 amplitude of the diffuse type was significantly reduced compared to that of NC. (**G)** The mean photopic a amplitude of the diffuse type was reduced compared to that of the intermediate type; however, no significant differences were found between the three types and the NC. (**H)** The mean photopic b amplitude of the diffuse type was significantly reduced compared to those of the other types and the NC. (**I)** The mean 30Hz N1P1 amplitude of the diffuse type was significantly reduced compared to those of the other types and the NC. Significant difference (P < 0.05) between each group versus controls or versus localized group or versus intermediate group was marked with * or † or ‡, respectively.

**Fig 3:**
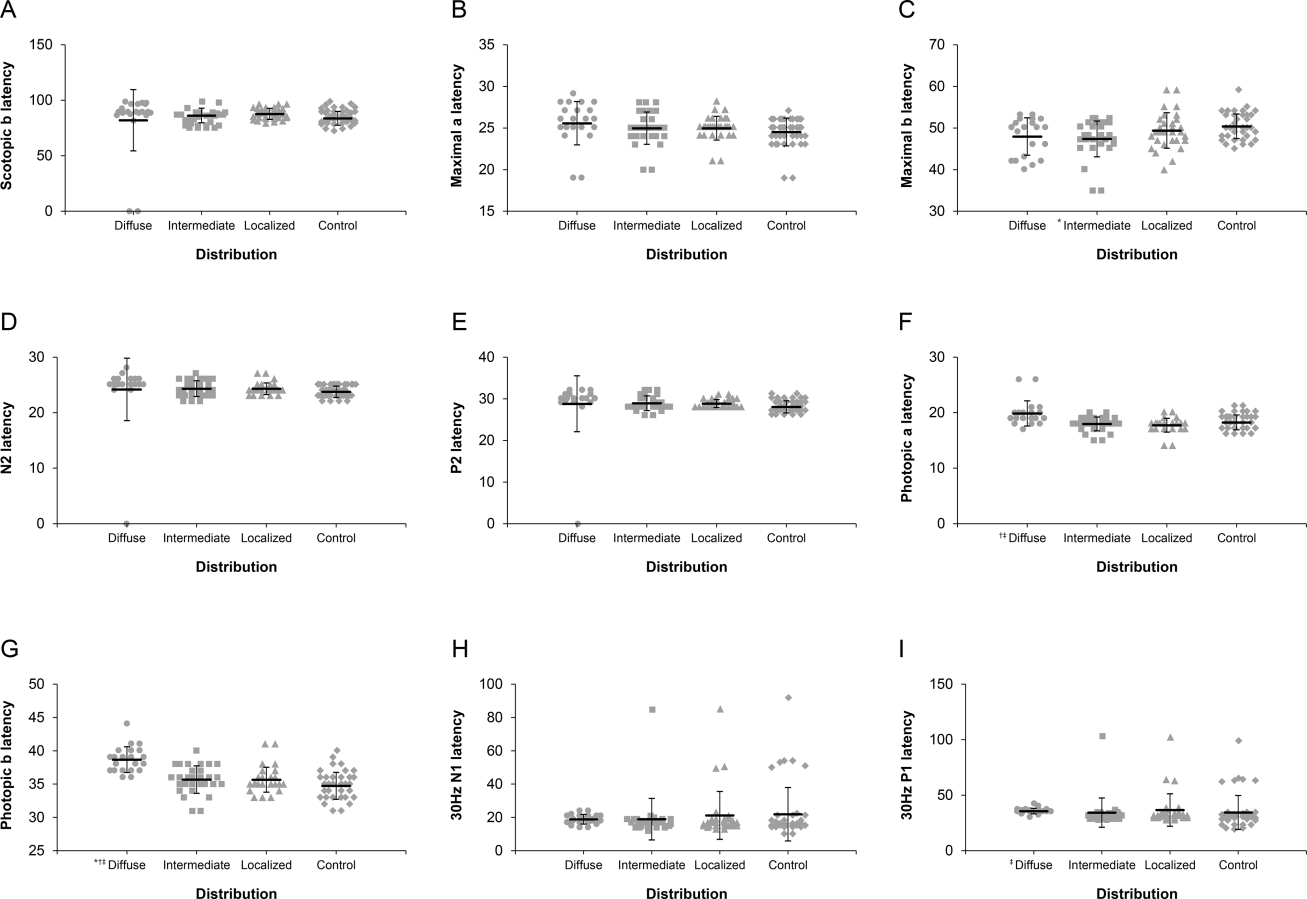
Comparison of electroretinogram (ERG) latencies among the three distribution types of reticular pseudodrusen (RPD) and the normal control (NC). No statistically significant differences were observed among the three types and the NC in the mean scotopic b latency (**A**), in the mean maximal a latency (**B**), in the mean N2 latency (**D**), in the mean P2 latency (**E**), and in the mean 30Hz N1 latency (**H**). (**C)** The mean maximal b latencies of the intermediate type were shorter than those of the NC. (**F)** The mean photopic a latency of the diffuse type was longer than those of the intermediate and localized type; however, no significant differences were found among the three types and the NC. (**G)** The mean photopic b latency of the diffuse type was longer than those of the other types and the NC. **I.** The mean 30Hz P1 latency of the diffuse type was longer than that of the intermediate type. Significant difference (P < 0.05) between each group versus controls or versus localized group or versus intermediate group was marked with * or † or ‡, respectively.

We compared ERG responses between the three distribution types and the control group (Figs 1–3) using age and sex adjustment method (Tables 3 and 4).

**Table 3:** Electroretinography (ERG) amplitudes of patients with reticular pseudodrusen and controls. (Statistical methods used were partial spearman correlation coefficient analysis with Bonferroni’s correction. Age and sex were adjusted, and age was restricted to over 50).

ERG Parameter	Diffuse RPD(D) Mean ± SD	Intermediate RPD(I) Mean ± SD	Localized RPD(L) Mean ± SD	Normal controls(NC) Mean ± SD	*P Value* (NC vs. D)	*P Value* (NC vs. I)	*P Value* (NC vs. L)	*P Value* (D vs. I)	*P Value* (D vs. L)	*P Value* (I vs. L)
Amplitudes (㎶)										
Scotopic Rod b wave amplitude	37.7±26.8	125.1±58.0	164.3±68.4	228.1±65.7	< .001*	< .001*	.005*	< .001*	< .001*	1.00
Maximal Combined a wave amplitude	123.4±39.8	204.8±69.3	237.8±70.4	269.4±77.2	< .001*	.97	1.00	< .001*	< .001*	1.00
Maximal Combined b wave amplitude	181.8±93.4	329.5±62.6	421.4±97.8	441.6±114.2	< .001*	.084	1.00	< .001*	< .001*	.056
Oscillatory Potential 1 amplitude	16.1±6.1	25.4±12.9	26.0±10.6	34.1±12.3	.005*	1.00	1.00	.21	.34	1.00
Oscillatory Potential 2 amplitude	36.1±15.5	67.4±23.0	84.0±29.7	95.3±31.1	< .001*	.59	1.00	.001*	< .001*	1.00
Oscillatory Potential 3 amplitude	11.9±7.1	22.8±21.1	24.3±15.4	27.2±13.3	.037*	1.00	1.00	1.00	.25	1.00
Photopic Cone a wave amplitude	35.3±12.1	52.9±18.4	50.9±14.9	55.2±16.9	1.00	1.00	1.00	.014*	.53	1.00
Photopic Cone b wave amplitude	73.0±23.7	133.1±48.1	139.0±41.3	152.2±33.4	< .001*	1.00	1.00	< .001*	< .001*	1.00
30Hz Flicker N1P1 amplitude	65.2±18.6	100.5±37.2	107.1±31.1	123.9±22.9	< .001*	1.00	1.00	.001*	< .001*	1.00

D: diffuse type; I: intermediate type; L: localized type; NC: normal control group; RPD: reticular pseudodrusen

* P value less than 0.05 were considered to be significant.

**Table 4:** Electroretinography (ERG) implicit times of patients with reticular pseudodrusen and controls. (Statistical methods used were partial spearman correlation coefficient analysis with Bonferroni’s correction. Age and sex were adjusted, and age was restricted to over 50.).

ERG Parameter	Diffuse RPD(D) Mean ± SD	Intermediate RPD(I) Mean ± SD	Localized RPD(L) Mean ± SD	Normal controls(NC) Mean ± SD	*P Value* (NC vs. D)	*P Value* (NC vs. I)	*P Value* (NC vs. L)	*P Value* (D vs. I)	*P Value* (D vs. L)	*P Value* (I vs. L)
Implicit Times (ms)										
Scotopic Rod b wave latency	82.0±27.6	86.2±6.6	87.6±5.0	83.7±6.2	1.00	1.00	1.00	1.00	1.00	1.00
Maximal Combined a wave latency	25.6±1.7	25.0±2.6	24.9±1.9	24.5±1.4	1.00	1.00	1.00	1.00	1.00	1.00
Maximal Combined b wave latency	48.0±4.5	47.4±4.3	49.4±4.3	50.4±3.0	.31	< .011*	1.00	1.00	1.00	1.00
Oscillatory potential N2 latency	24.2±5.6	24.3±1.4	24.3±1.0	23.8±1.0	1.00	1.00	1.00	1.00	1.00	1.00
Oscillatory potential P2 latency	28.8±6.7	28.9±1.8	28.9±1.0	28.0±1.4	.57	1.00	1.00	.75	1.00	1.00
Photopic Cone a wave latency	19.9±2.3	18.0±1.2	17.7±1.2	18.2±1.3	1.00	1.00	1.00	.026*	.003*	1.00
Photopic Cone b wave latency	38.7±1.9	35.7±2.1	35.6±1.9	34.7±2.0	< .001*	1.00	1.00	< .001*	< .001*	1.00
30Hz Flicker N1 latency	18.9±2.9	18.9±12.5	21.2±14.4	21.9±16.0	1.00	1.00	1.00	1.00	1.00	1.00
30Hz Flicker P1 latency	35.6±2.5	34.3±13.2	36.7±14.6	34.4±15.3	.48	1.00	1.00	.022*	1.00	1.00

D: diffuse type; I: intermediate type; L: localized type; NC: normal control group; RPD: reticular pseudodrusen

* P value less than 0.05 were considered to be significant.

The mean amplitudes of scotopic rod response, maximal combined response, OP, photopic cone response, and 30Hz cone flicker response were more decreased in the diffuse, intermediate, and localized type, in order, except for the photopic cone a-wave response, in which the intermediate type showed more increased response than the localized type (Fig 2, Table 3).

The mean amplitudes of the diffuse type showed a statistically significant decrease compared with those of the control group under all testing conditions, except for the photopic a-wave response. However, the mean amplitudes of ERG responses of the intermediate and localized type showed no statistical differences from those of the control group in all tests, except for the scotopic b-wave response. The mean amplitudes of ERG response showed statistical differences between the diffuse type and the other two types under all testing conditions, except for OP1 and OP3 and photopic cone response. However, there were no statistical differences between the intermediate type and the localized type under all testing conditions (Fig 2, Table 3).

The mean implicit times of ERG responses were not significantly delayed in RPD eyes compared to eyes in the control group under most testing conditions, except for the photopic b-wave (Fig 3, Table 4). The mean implicit time of diffuse type was delayed compared to those of other two types in the photopic a-wave and the photopic b-wave. The mean implicit time of diffuse type was delayed compared to those of the intermediate type in the 30 Hz flicker P1 (Fig 3, Table 4).

## Discussion

Although RPD were first described 20 years ago, consensus has been elusive on the precise meaning of the term and the standard diagnostic criteria. The term “reticular drusen” was used by Klein et al[13] in the Wisconsin age-related maculopathy grading system, and the term “reticular pseudodrusen” was used by Arnold et al,[5] in the absence of drusenoid deposit material in one histologic specimen, which probably was a preparation artifact. Recently, Zweifel et al[4] proposed the use of the term “subretinal drusenoid deposit” rather than “reticular pseudodrusen” based on SD OCT and histological findings. Smith et al[8] even used the term “reticular macular disease” as a clinical disease entity characterized by the presence of RPD. In this report, we use the term reticular pseudodrusen not because we think that it is correct, but because there is still no generally accepted term. The distributional and functional characteristics of RPD may be helpful in providing the right term.

Detection of RPD have varied among studies. These include a single test[1, 13, 14] or a combination of multiple tests,[2, 4–8, 15] including fundus color photography with or without blue channel examination, near infra-red imaging, red-free imaging, FAF imaging, ICGA, and SD OCT. Among them, near infra-red imaging, ICGA, and SD OCT were reported to be the most sensitive for the detection of RPD.[3, 4, 8, 15] In this article, we used more than two methods in most patients (92.7%) for diagnosis of RPD in order to avoid possible misdiagnosis, and all patients received color fundus photography and blue channel examinations. In addition, four-fifths of the patients had both high resolution SD OCT and infra-red imaging tests. We think that our methods of diagnosis of RPD were appropriate and reliable, because we used multiple imaging modalities, including the most sensitive detection methods, and decisions were made according to agreement of two retinal specialists.

With the help of recent advancements in imaging technologies, RPD have been revealed to have several different features from soft drusen usually seen in AMD. Distinguishing features include the distribution pattern in the fundus and the location of deposits. However, much is still unknown about RPD, such as the incidence, prevalence, age of initial onset, fundus distribution in extra-macular areas, natural course, progression, retinal function, complications, and visual prognosis. The type or extent of fundus distribution might be clinically important for diagnosis, staging, and prognosis of RPD, because the diffuse type showed far less electrophysiological function than the localized type. In addition, any change of distribution could provide clues on the progression or regression of RPD.[15] We previously evaluated the distribution of RPD, and the prevalence of accompanying AMD. The prevalence of accompanying late AMD was 13.9%, 13.8%, 56.7%, in the localized, intermediate, and diffuse distribution type, respectively, and was significantly higher in the diffuse type than in other types (P<0.05).[9] Therefore, we think that it is necessary to examine both the macular area and the extramacular or peripheral fundus area in every RPD eye, combined with the appropriate functional tests.

We used full-field ERG to evaluate whole retinal function in RPD, and the ERG amplitudes of the diffuse type were more decreased than those of the other types and the control group under most testing conditions. Alten et al. reported no definite influence on electrophysiologic activity in retinal areas affected with RPD using multifocal ERG (mfERG) in eyes with RPD.[10] However, the participants were patients with RPD in the posterior pole. In the current study, the extent of reduction in the ERG amplitudes of many testing conditions were significantly associated with the distribution types. Interestingly, ERG under photopic conditions showed significantly reduced amplitudes only in the diffuse type compared with controls except photopic a amplitude. Under scotopic condition, however, amplitude decreased significantly in all three RPD distribution types compared with controls. The data are supported by that presence of RPD and thin choroid were associated with impairments in dark adaptation.[12] They are also consistent with previous articles, which revealed that RPD were frequently localized to the perifovea where rod showed a high density in histologic analysis, and rod function was more severely affected than cone function in microperimetry.[16, 17] In the follow-up study, Alten et al. also reported decline of retinal function over time in eyes with progressive RPD.[18] The collective results support the speculation that RPD are generalized, perhaps progressive retinopathy, and not merely macular diseases. Eyes with RPD progression showed outer retinal atrophy and loss of underlying choroidal thickness including a decrease in the photoreceptor length.[11]

Reduced amplitudes in ERG may reflect a general reduction of functional retinal activity by several factors other than pathologic changes of RPD.[19] Electrophysiological retinal function can be decreased with age[20, 21] and the effect of accompanying AMD[22, 23]. In previous reports on effect of aging on ERG responses, the amplitude was decreased linearly; however, the prolongation of implicit times was controversial[19, 20]. Similar to the aging effect, RPD appeared to have a strong effect on the amplitude; however, the effect on implicit time was various and less than that on the amplitude. Although we could not match age, statistical analysis using the age adjustment method suggested that the decreased responses of ERG were more associated with RPD themselves than the aging effect. There have been several reports of reduced and delayed ERG responses in AMD.[22, 23] However, the proportion of eyes having RPD with AMD was not different among three different RPD distribution types, although ERG responses showed differences in comparison among types. Furthermore, unlike in RPD, scotopic a- and b-waves of the ERG were revealed mildly affected in eyes with AMD. Thus, the impaired ERG responses were more related with RPD themselves than accompanied AMD. Other factors reported to affect ERG amplitude and implicit time included gender, refractive error, intraocular pressure, and pigmentation.[19] The effect of gender was reported to be small; amplitudes were slightly smaller in males than in females, possibly due to the greater axial length in males. The majority of RPD patients were female, and statistical analysis using sex adjustment suggested that the decreased responses of ERG were more associated with RPD themselves than the gender effect; therefore, the gender effect on reduction of amplitude appeared to be negligible in this study. The effects of refractive error, intraocular pressure, and pigmentation were not presently evaluated. However, there were no patients with high myopia, severe lens opacities, or glaucoma with severe visual field loss.

Our data suggest that RPD affects the function of rod as well as cone photoreceptors, and that the function of bipolar and amacrine cells are also affected. Recently, a study using multispectral confocal scanning laser ophthalmoscopy (MS cSLO) showed progression of RPD into the photoreceptor layers more sensitively than previous modalities.[24] Further studies with various modalities including MS c SLO are needed to investigate the basic mechanism of functional deterioration.

Our study has limitations, including inherent biases in the third referral hospital, the normal control group comprised of age, sex, and number unmatched persons, patients who did not undergo the same diagnostic imaging tests, patients who showed not only RPD but accompanied AMD, a retrospective case series with various underlying diseases, and the non-randomized, non-prospective, and not well controlled study design.

## Conclusion

ERG responses were more reduced in the diffuse RPD distribution type, illustrating that retinal functional abnormalities were related to the size of the retinal area affected with RPD. Although well-designed, quantified, prospective studies are needed to confirm the relationship between distribution and retinal function, fundus examination of both macular and extramacular areas, or the retinal functional test should be considered in the study design and analysis of future RPD studies.

## Supporting information

S1 Fig Fundus montages of 3 distribution types. (A) Localized distribution. Reticular pseudodrusen (RPD) are observed in the central field and less than 1/3 area of superior and temporal photographic fields. (B) Intermediate distribution. RPD are observed in the central field, more than 1/3 area of superior field, and less than 1/3 area of temporal field. (C) Diffuse distribution. RPD are observed in the central field and more than 1/3 area of all 4 adjacent fields taken by the protocol. (Reprinted with permission from Lee MY, Yoon J, Ham D-I: Clinical features of reticular pseudodrusen according to the fundus distribution. Br J Ophthalmol 2012 Sep;96(9):1222–6. Copyright BMJ Publishing Group LTD.)
(TIF)

S1 Appendix Clinical characteristics and ocular measurements data of all subjects. (XLS)
