# Supplementary figures and images for: Electrophysiological function in eyes with reticular pseudodrusen according to fundus distribution
Source: PLoS One. 2018 Aug 29;13(8):e0203146. doi: 10.1371/journal.pone.0203146 (PMC6114915; doi:10.1371/journal.pone.0203146)

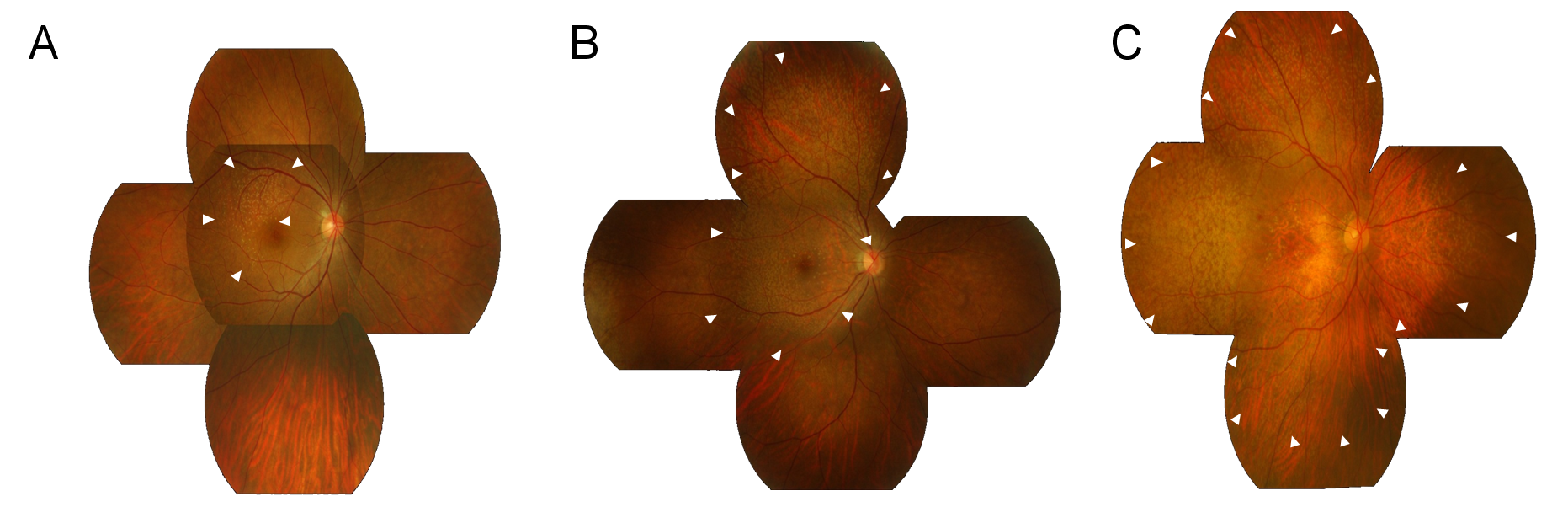

Supplement: S1 Fig — (A) Localized distribution. Reticular pseudodrusen (RPD) are observed in the central field and less than 1/3 area of superior and temporal photographic fields. (B) Intermediate distribution. RPD are observed in the central field, more than 1/3 area of superior field, and less than 1/3 area of temporal field. (C) Diffuse distribution. RPD are observed in the central field and more than 1/3 area of all 4 adjacent fields taken by the protocol. (Reprinted with permission from Lee MY, Yoon J, Ham D-I: Clinical features of reticular pseudodrusen according to the fundus distribution. Br J Ophthalmol 2012 Sep;96(9):1222–6. Copyright BMJ Publishing Group LTD.) (TIF) [file pone.0203146.s001.tif]
